# Thymoquinone-Loaded Polymeric Films and Hydrogels for Bacterial Disinfection and Wound Healing

**DOI:** 10.3390/biomedicines8100386

**Published:** 2020-09-28

**Authors:** Anika Haq, Suneel Kumar, Yong Mao, Francois Berthiaume, Bozena Michniak-Kohn

**Affiliations:** 1 Department of Pharmaceutics, Ernest Mario School of Pharmacy, Rutgers, The State University of New Jersey, Piscataway, NJ 08854, USA; mimanikahaq@gmail.com; 2 Center for Dermal Research, Life Sciences Building, Rutgers, The State University of New Jersey, Piscataway, NJ 08854, USA; 3 Department of Biomedical Engineering, Rutgers, The State University of New Jersey, Piscataway, NJ 08854, USA; sk1350@soe.rutgers.edu (S.K.); fberthia@soe.rutgers.edu (F.B.); 4 Department of Chemistry and Chemical Biology, Rutgers, The State University of New Jersey, Piscataway, NJ 08854, USA; maoy@dls.rutgers.edu

**Keywords:** thymoquinone, polymeric film and hydrogel, topical/transdermal drug delivery, wound disinfection, *Staphylococcus aureus*, bacterial skin infections

## Abstract

The purpose of this study was to synthesize and characterize novel biocompatible topical polymeric film and hydrogel systems that have the potential to deliver the antibacterial agent thymoquinone (TQ) directly to the skin target site to manage the local wound infection and thereby wound healing. The polyvinyl pyrrolidone (PVP) matrix-type films containing TQ were prepared by the solvent casting method. In vitro skin permeation studies on human cadaver skin produced a mean flux of 2.3 µg TQ/cm^2^/h. Human keratinocyte monolayers subjected to a scratch wound (an in vitro wound healing assay) showed 85% wound closure at day 6 in the TQ group (100 ng/mL TQ) as compared to 50% in the vehicle control group (*p* = 0.0001). In a zone-of-inhibition (ZOI) assay, TQ-containing films and hydrogels completely wiped out *Staphylococcus aureus* in 10 cm diameter Tryptic Soy Agar plates while 500 µg/mL gentamicin containing filters gave 10 mm of ZOI. In an ex vivo model, TQ-containing films eradicated bacterial colonization on human cadaver skin. Furthermore, in a full-thickness wound infection model in mice, TQ-containing films showed significant activity in controlling *Staphylococcus aureus* infection, thereby disinfecting the skin wound. In summary, TQ-containing PVP films and hydrogels developed in this study have the potential to treat and manage wound infections.

## 1. Introduction

The skin is the largest organ in the body and occupies about 16% of the total body weight of an adult and has a surface area of about 2 m^2^ [1]. It is a complex arrangement of structures and has a multifunctional role—provide a physical barrier to the environment by acting as a protective barrier against the ingress of foreign material, maintains homeostasis and thermoregulation by limiting the loss of water, electrolytes, and heat and prevents microbial colonization [2,3]. Although skin act as a shield against bacterial invasion, bacteria can still invade the epidermis and dermis to produce localized infections and cause a variety of pathologic changes in the skin (impetigo, furuncles, subcutaneous abscesses) [4]. Microbial infections of the skin and underlying tissues are among the most frequent conditions found in ambulatory care patients [5]. *Staphylococcus aureus* (*S. aureus*) is one of the most important human and veterinary pathogens and is the causative agent for the majority of primary skin infections [6]. It causes infections ranging from benign to life-threatening diseases [7]. Skin and soft tissue infections (SSTIs) encompass a wide variety of clinical outcomes, ranging from mild cases of cellulitis, erysipelas, trauma, subcutaneous tissue infections, wound-related infections to complicated deep-seated infections with a systemic sign of sepsis [8]. SSTIs may lead to severe complications and hospital admission when associated with co-morbidities and/or bacteremia. The most commonly reported cause of SSTIs is *S. aureus* followed by β-hemolytic streptococci (BHS) [9,10]. The *S. aureus* can internalize by a variety of nonphagocytic host cells and can contribute to the development of persistent or chronic infections and may lead to deeper tissue infections or dissemination [11,12,13].

Wound management is a prevalent clinical problem as wound healing involves a series of complex processes including the inflammation phase, proliferative phase (formation of granulation tissue, re-epithelialization, and matrix formation), and remodeling phase [14]. Each phase of wound healing is well defined, although they overlap with the next [15]. The process of wound healing becomes delayed when wounds are colonized, and the colonizing agent is sustained [16]. In the patient with a weak immune system, bacterial contamination can prolong wound healing [17,18] and colonization of bacteria in wounds is a serious threat. Open wounds are also at high risk of invasive wound infections, which can further lead to amputation and disability [19]. SSTIs and wound healing all rely on efficient antibiotic therapies. Temporary eradication of *S. aureus* with antibiotics often leads to clinical improvement. Antimicrobial resistance is one of the biggest challenges in the global health sector [20]. The high incidence of methicillin resistance in hospitals complicates the prevention and treatment of serious infections due to staphylococci [21]. As infections due to multi-resistant Gram-positive organisms are increasing day by day, their early recognition, treatment, and proper management are greatly required. Several antimicrobial agents in different dosage forms are available for the treatment of SSTIs. Topical application of antibiotic agents have several benefits over oral and systemic therapy [22]—localized and targeted delivery can provide the required concentration for antibiotic activity more efficiently at the skin target site, can avoid unnecessary exposure of gut flora that may exert selection for resistance, can avoid side-effect and allergic reactions associated with systemic antibiotic treatment. Therefore, topical applications may highly influence the treatment efficiency and can increase patient compliance.

In such a scenario, the development of a new treatment strategy is crucial to deal with the emerging issues of skin infections. The topical delivery of antibacterial agents of medicinal plants can be considered as a source for new therapeutic agents aimed at the treatment and management of skin infections. Thymoquinone (TQ, 2-isopropyl-5-methyl-1,4-benzoquinone) is the main constituent of *Nigella sativa* (Black cumin) seeds [23]. TQ is a yellow crystalline molecule and has a basic quinone structure consisting of a para-substituted dione conjugated to a benzene ring to which a methyl and an isopropyl side chain groups are added in positions 2 and 5, respectively. TQ has many pharmacological properties such as anticancer, antimicrobial, anti-inflammatory, antioxidant, anti-asthmatic, and immunomodulatory effects [24]. Thus far, several research groups have reported the compound’s anticancer and brain targeting properties. Odeh et al. loaded TQ in a liposome system and tested on breast cancer cell lines (MCF-7 and T47D) to evaluate TQ anticancer properties [23], Ng et al. prepared TQ loaded nanostructured lipid carrier and showed its effectiveness towards breast cancer and cervical cancer cell lines [25], and Ahmad et al. evaluated TQ-loaded mucoadhesive nano-emulsion for the treatment of cerebral ischemia [26]. However, there have been no reports on TQ delivery from a topical delivery system and its effectiveness for the treatment of wound and *S. aureus* associated bacterial skin infections.

The objective of this study was to synthesize and characterize a biocompatible novel topical polymeric film and hydrogel system that has the potential to deliver antibacterial TQ agent directly at the skin target site that may be useful for the treatment and management of *S. aureus* related bacterial skin infections and for the wound management. To achieve this objective, TQ loaded polyvinyl pyrrolidone (PVP) films were prepared using a solvent casting method and TQ wound hydrogels were prepared using different polymers. The prepared films and hydrogels were characterized by physical parameters, permeability, and stability studies. Its biocompatibility was assessed, and the antibacterial efficacy of films and hydrogels were evaluated in vitro and ex vivo on selected strains of *S. aureus*. Further, in vitro scratch assay models using human dermal fibroblast (HDF) and human keratinocyte cell line (HaCaT) were used to demonstrate its wound healing properties. To evaluate its preclinical and in vivo efficacy, a biopsy punch wound infection animal model was used. This work demonstrates that the PVP/TQ film is effective in the control of bacterial infection and facilitating wound healing.

## 2. Materials and Methods

### 2.1. Materials

Thymoquinone (TQ), polyvinylpyrrolidone (PVP), dibutyl phthalate (DBP), hydroxypropyl methylcellulose (HPMC), potassium chloride, benzoic acid, gentamicin solution, formalin solution (10%), triethanolamine, propylene glycol (PG), dipropylene glycol (DiPG), alamarBlue^®^ (resazurin) assay kit, high-performance liquid chromatography (HPLC) grade water and acetonitrile were purchased from Sigma-Aldrich Co. (St. Louis, MO, USA). Laurocapram (Azone) was purchased from BOC Sciences (Shirley, NY, USA). Phosphate-buffered saline tablets (PBS, pH 7.4) was purchased from MP Biomedicals, LLC (Solon, OH, USA), ethanol (EtOH) was purchased from Decon Labs, Inc. (King of Prussia, PA, USA), xanthan gum (XG) was purchased from Spectrum Chemical (New Brunswick, NJ, USA), hydroxypropyl cellulose (HPC) was purchased from Ashland (Wilmington, DE, USA). Carbopol 980 and ultrez 10 were purchased from Lubrizol (Cleveland, OH, USA) and drierite was purchased from Acros organics (Morris Plains, NJ, USA). Bacto tryptic soy broth, bacto agar, Dulbecco’s Modified Eagle’s Medium (DMEM), and Dulbecco’s phosphate-buffered saline (DPBS) were purchased from Fisher Scientific (Hampton, NH, USA). Human HaCaT cell line, HDF, and Pen/strep were purchased from Life Technologies (Carlsbad, CA, USA). *S. aureus* (ATCC 49230) was purchased from ATCC (Manassas, VA, USA). Isoflurane was purchased from Henry Schein (Dublin, OH, USA). Fetal bovine serum (FBS) was purchased from Atlanta Biologicals (Minneapolis, MN, USA). CellTiter 96^®^ was purchased from Promega (Madison, WI, USA). Gentamicin sulfate cream USP, 0.1% was purchased from Perrigo (Allegan, MI, USA). Dermatomed human cadaver skins from the posterior torso were obtained from New York Firefighter Skin Bank (New York, NY, USA). Adult male BALB/c mice (9 weeks old) were purchased from Charles River (Wilmington, MA, USA).

### 2.2. Fourier Transform Infrared (FTIR) Analysis

FTIR spectra of samples were taken on a model Nicolet iS10 instrument Thermo Scientific (Waltham, MA, USA) to investigate the possible interaction between the drug and polymer. FTIR spectra of pure drug, polymer, physical mixture of drug and polymer in a ratio of 1:1, films with TQ and films without TQ were scanned in the range between 400 and 4000 cm^−1^.

### 2.3. Fabrication of Films

The matrix-type polymeric films containing TQ were prepared by the solvent casting method. Accurately weighted TQ (1%, *w*/*w*) was dissolved in ethanol and was sonicated for 30 min to ensure solubilization. DBP was used as a plasticizer and Azone was used as a penetration enhancer. The weighted amount of PVP, DBP (4%, *w*/*w*), and Azone (5%, *w*/*w*) was added in the drug solution. The mixture was stirred at 200 rpm at 25 °C for 20 min. The solution was poured on a Teflon dish (15 cm^2^) and placed in an oven maintained at 60 ± 5 °C. To allow complete evaporation, the system was left undisturbed for 3-h and 20 min. The formed films were completely removed from the Teflon dish and punched out into 0.64 cm^2^ pieces. Control films were prepared without TQ but containing PVP, plasticizer, and penetration enhancer.

### 2.4. Preparation of TQ Hydrogel Formulations

Drug-loaded hydrogels were prepared using gelling agents, preservatives, penetration enhancers, and vehicles. Different concentrations of various polymers (gelling agents) with or without xanthan gum were dispersed slowly in an aqueous-based solution containing TQ (0.2% *w*/*w*), 1:1 concentration of propylene glycol, and dipropylene glycol (20% *w*/*w*, as a vehicle), benzoic acid (0.1% *w*/*w*, as a preservative), ethanol (5% *w*/*w*, as a penetration enhancer), using an overhead mechanical stirrer at a moderate speed. Triethanolamine was used to adjust the pH of Carbopol and Ultrez 10. The prepared hydrogels were packed in a wide-mouth jar covered with screw-capped plastic lid and kept in dark and at laboratory ambient temperature. The composition of different prepared TQ hydrogel formulations is given in Table S1.

### 2.5. Field Emission Scanning Electron Microscopic (FESEM) Studies

The surface morphology of the film was recorded with a Zeiss field emission scanning electron microscopy (FESEM) (FSD PRE-AMP 4CH, Oberkochen, Germany). The film sample was mounted on an aluminium stub with the double-sided adhesive band then gold was sputtered on the specimen (20 nm) to ensure sufficient electrical conductivity. An accelerating voltage of 5 kV was applied, and the image was photographed by a secondary electron detector.

### 2.6. Physicochemical Characterization of Films

#### 2.6.1. Film Thickness

Film thickness was measured using a digital caliper (Fisher Scientific, Portsmouth, NH, USA) at three different places, and the mean value was calculated.

#### 2.6.2. Drug Content Uniformity

A prepared film was dissolved in 10 mL ethanol and stirred continuously for 24 h. The drug content was analyzed using the HPLC method described in Section 2.10.

#### 2.6.3. Weight Variation

Weight variation was studied by individually weighing five randomly selected films.

#### 2.6.4. Flatness

Each film was cut into three sections (left, center, and right). The length of each section was measured to examine the variation in length that can arise from nonuniformity in flatness and was calculated as a percentage of constriction, with 0% constriction equivalent to 100% flatness.

% Constriction = [(L1 − L2)/L2] × 100

where L1 = the initial length of each strip and L2 = the final length of each strip.

#### 2.6.5. Folding Endurance

To determine the folding endurance the film was folded repeatedly at the same place until it broke. The folding endurance value represents the number of times the film could be folded at the same place without breaking. 

#### 2.6.6. Percentage of Moisture Content

The prepared films were marked, then weighed individually and kept in a desiccator containing drierite at room temperature for 24 h. The individual weights of the films were measured from time to time until a constant weight was observed. The difference between initial and final weight with respect to final weight was used to measure the percentage of moisture content. 

#### 2.6.7. Percentage of Moisture Uptake

The films were weighed and kept in a desiccator at room temperature for 24 h. Films were taken out and placed in a desiccator containing 100 mL of a saturated solution of potassium chloride to maintain 84% relative humidity until a constant weight for the films were obtained. The percentage of moisture uptake was calculated as the difference between final and initial weight with respect to the initial weight.

### 2.7. Physicochemical Characterization of the Prepared Hydrogels

#### 2.7.1. Visual Inspection

TQ hydrogels were examined visually for their color and homogeneity (appearance and presence of any aggregates).

#### 2.7.2. pH Determination

The pH of various TQ hydrogel formulations was determined using pH meter (Symphony B10P pH meter, VWR, Radnor, PA, USA). One g of TQ hydrogel was dissolved in 10 g of DI water. After 2 h pH was determined at room temperature.

#### 2.7.3. Spreadability Test

A 10 mg sample was placed on top of a microscopic slide and covered with another slide, 50 gm of standardized weight was put on it and after 1 min the diameter of the sample was taken in mm.

#### 2.7.4. Drug Content Uniformity

Three samples of a specific quantity (100 µL) of each prepared hydrogel were taken and dissolved in 10 mL of ethanol solvent. To ensure drug solubility the 20 mL glass vial containing the gel solution was put on a magnetic stirrer at 600 rpm at 25 °C overnight. The drug content was then determined using HPLC. The variability of TQ content in hydrogels was reported as % RSD:

% RSD = (standard deviation/mean drug content) × 100

### 2.8. Rheological Characterization of the Hydrogel Formulation

Rheological characterization was performed on a Malvern Kinexus Ultra + rheometer (Malvern, Worcestershire, UK) equipped with a 25 mm flat stainless-steel plate. All tests were done at 32 °C and a gap of 1 mm. The following tests were carried out:

#### 2.8.1. Oscillation Stress Sweep

The samples were subjected to increasing stress (0.1–500 Pa) at a constant frequency of 1 Hz. This test allows determination of the linear viscoelastic region (LVR) of the sample, and therefore the consequent choice of the stress value to use in the subsequent oscillation test.

#### 2.8.2. Frequency Sweep

All the prepared samples were subjected to an increasing frequency of 0.1–50 rad/s at constant stress (5 Pa) obtained from LVR. The effect of stress on elastic modulus (G′) and viscous modulus (G′′) was monitored.

### 2.9. In Vitro Skin Permeation Studies

Franz diffusion cell (FDC, Permegear Inc., Hellertown, PA, USA) and dermatomed human cadaver skin (~500 µm thickness) were used to perform in vitro skin permeation studies. Skin samples were prepared by slowly thawing at room temperature, cutting into appropriate pieces, and then soaking in filtered PBS (pH 7.4) for 15 min. After that, they were mounted on FDC (0.64 cm^2^ donor area and receptor volume of 5.0 mL) with the epidermal side in contact with the formulation or donor compartment. Filtered PBS (pH 7.4) was used as a receptor solution to fill the receptor compartment of each cell. The cell temperature was maintained at 37 °C under synchronous continuous stirring using a magnetic stirrer at 600 rpm and left for 15 min to equilibrate the diffusional membranes. At time zero, formulated films and 100 µL of hydrogel were placed over the skin in the donor compartment of each cell. At each time point, 300 µL of receptor samples was withdrawn from the sampling port and replaced with an equal amount of receptor solution. The receptor aliquots of 300 µL were analyzed at the end of the experiment using a valid HPLC method described below.

### 2.10. High-Performance Liquid Chromatography (HPLC)

For this study, an Agilent 1100 series instrument (Agilent Technologies, Santa Clara, CA, USA) coupled with UV detection (DAD) and HP Chemstation software V. 32 was used to validate the HPLC method. To analyze the concentration of TQ, a mobile phase of 80% acetonitrile and 20% water was pumped through an Agilent Eclipse XDB-C18 5 µm, 250 × 4.6 mm column with an injection volume of 20 µL and flow rate of 1.0 mL/min. The column temperature was set to 23 °C with UV detection of 250 nm used, with a retention time of 4.2 min. At a concentration of 0.39–100 µg/mL, the method was linear with an R^2^ value of 0.99. Intra- and inter-day precision and accuracy of the method showed a % CV of 0.01 and 0.1, respectively, which is lower than the requirements of 2%.

### 2.11. Skin Deposition Study

At the end of the permeation study, the skin was removed from the diffusion cell. The skin samples were then cut around the diffusional area, air-dried, accurately weighed, and placed into bead bug tubes. To extract the drug, the skin samples inside the tube were cut into very small pieces using a pair of scissors, and 1 mL ethanol was added to each tube. These were then homogenized for 9 min (3 min of 3 cycles) using a BeadBug^TM^ Microtube homogenizer, D1030 (Benchmark Scientific, Sayreville, NJ, USA). All the skin samples were agitated at 37 °C for 24 h using a Julabo SW22 shaker (Julabo USA Inc., Allentown, PA, USA). After that, they were centrifuged at 1200 rpm for 5 min, and each of the skin samples was filtered through a 0.45 µm polypropylene filter medium with polypropylene housing. TQ concentrations were expressed as ng of TQ per skin weight in mg.

### 2.12. Stability Study

The films were put in a Petri dish and the dish was wrapped by aluminum foil and stored at 20 °C for 60 days. The samples were analyzed for physical changes such as color, texture, and other physical parameters. The FTIR spectra of stored films were compared with freshly prepared films. The films were also analyzed for drug content. On the other hand, all the hydrogel formulations were filled in glassware and covered with aluminum foil and were kept at laboratory ambient temperature for 8 months. The physical stability of the formulation was examined visually for appearance, color, and odor in every two weeks. After 8 months an antibacterial efficacy study was performed to confirm the formulation stability of hydrogel formulation.

### 2.13. In Vitro Antibacterial Activity of TQ Films and Hydrogels

The prepared control films and TQ loaded films were tested for their antibacterial activity against *S. aureus* (ATCC 49230) using the disc diffusion method. Briefly, Muller Hinton agar (MHA) plates were used for screening, prepared by pouring 15 mL of molten media into sterile Petri dishes. Then 150 µL of overnight cultured bacteria adjusted to OD concentration of 0.602 (OD 1 = 1 × 10^9^/mL of bacteria) in sterile TSB (Tryptic soy broth) was spread on the surface of MHA agar plates with the help of a sterile spreader. The disc-shaped polymer film of 0.64 cm^2^ and 100 µL of TQ gel were then placed on the surface of the medium and incubated at 37 °C for 24 h. Gentamicin 500 µg/mL and 50 µg/mL was used as a positive control, UV irradiated filter paper was used as a negative control, and control film without TQ was used as a control. At the end of incubation, the inhibition zones were examined around the polymer disc films. The study was performed in triplicate.

### 2.14. Ex Vivo Antibacterial Activity of TQ Films and Hydrogels Using Human Cadaver Skin Explants

Human cadaver skin was thoroughly washed three times using sterile PBS. Using surgical gloves and sterile scissors they were cut into pieces (2 cm × 2 cm) and two skin samples were placed on each agar plate. 5 µL of 1 × 10⁶ CFU/mL was put onto each skin piece followed by the application of treatment. After overnight incubation at 37 °C, bacteria were extracted for counting using sterile PBS and 10 s of vortex. Serial dilutions of bacteria were prepared and were plated on TSB agar plates. Bacteria were counted after overnight incubation at 37 °C.

### 2.15. Cytocompatibility Study

AlamarBlue^®^ (resazurin) assay was used to evaluate the cytocompatibility of the TQ film using two cell lines, HaCaT (passage 8) and HDF (passage 5). The cells were counted and were seeded into the 6 well plates at a density of 200,000 cells/cm². After reaching confluency, the cells were treated with the samples for 24 h. Cells treated with media containing 1% Triton served as positive control and cells in media without any treatment acted as a negative control. After 24 h the cells were treated with alamarBlue^®^ and incubated for 4 h. The optical density was measured at an excitation-emission wavelength of 560–590 nm using Spark 10 M multimode microplate reader (Tecan, Männedorf, Switzerland). The percentage of cell viability was calculated using the formula given below:

% Cell viability = [(Fluorescent intensity)_test_/(Fluorescent intensity)_control_] × 100

### 2.16. Scratch Assay for Wound Closure Activity

The HDF (passages 2–4) cells were counted and were seeded into the 24-well plates at a density of 50,000 cells/well. After reaching confluency, the culture media was replaced with sterile base media (DMEM with 1% *P*/*S*), and scratch wounds were created in the cell monolayer using a 200 µL sterile pipette tip [27]. TQ in DMEM without serum at different concentrations (1 ng/mL and 100 ng/mL) was put into the culture media and the experiment was continued for 24 h. Base media was used as a control. Images were taken at 0, 4, 8, 12, and 24 h. The HaCaT (passage 37–39) cells were counted and were seeded into the 24 well plates at a density of 250K cells/well. After reaching confluency, the culture media was replaced with sterile base media (DMEM with 1% FBS + 1% *P*/*S*), and scratch wounds were created in the cell monolayer using a sterile pipette tip. TQ in DMEM at different concentrations (1 ng/mL and 100 ng/mL) was put into the culture media and the experiment was continued for six days. Base media was used as a control. Images were taken at 0, 24, 48, 72, and 144 h.

### 2.17. In Vivo Bacterial Skin Infection Study

**Figure 1:**
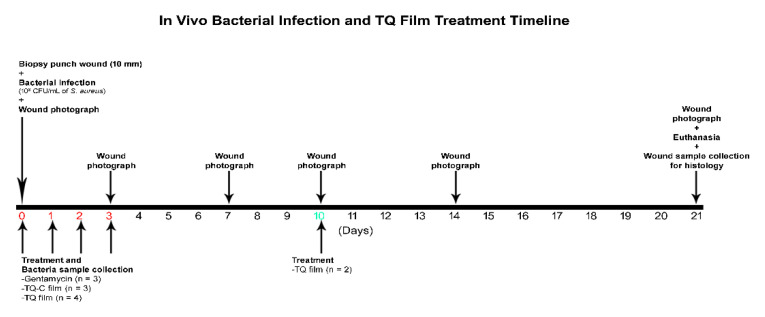
Flow chart depicting the experimental design for in-vivo evaluation of TQ film in a mouse model of bacterial infection. The number of mice used in each experimental group and the timeline for treatment. The time of treatment and sample collection of bacteria (red letters) is shown for each group (green letter indicates on TQ film treatment on day 10).

Animal infection experiments were performed at the Nelson Biological Laboratories, Rutgers University (Piscataway, NJ, USA) following a protocol approved by the Rutgers University Institutional Animal Care and Use Committee (IACUC ID: PROTO201702583, approved on 24 September 2019). Figure 1 shows the in-vivo experimental design. Briefly, mice were housed under standard conditions of light and temperature and were fed a standard diet and water ad libitum. Adult male mice (BALB/c, 10 weeks) were used for all experiments after habituating for a week at laboratory conditions [28,29]. Before the experiment day, mice were anesthetized using 5% isoflurane and then maintained at 2–3% during the procedure. The hair over the dorsum (head to tail) was shaved with an electric clipper first and then the remaining hair was removed with a depilatory cream (Nair^TM^ hair remover lotion, Church & Dwight, Ewing Township, NJ, USA). Finally, the shaved area was washed with wet scrub, and the animals were returned to their cages. On the next day, a 10 mm biopsy punch was used to create the full-thickness wound on the dorsum of the mice. Immediately afterward bacterial infection at the wound site was initiated by placing a 10 µL droplet containing 10^8^ CFU/mL of *S. aureus* from an overnight bacterial culture in the stationary phase. Mice were divided into the following groups namely Control wound (10 mm biopsy skin wound), Bacterial wound (skin wound infected with bacteria), Control film (wound infected with bacteria and then treated with control film without TQ), TQ film (wound infected with bacteria and then treated with TQ loaded film), and Gentamicin (wound infected with bacteria and then treated with gentamicin marketed cream formulation). Films with or without TQ were applied at the wound infection site on Day 0, 1, 2, and 3 (only TQ film, *n* = 2). Gentamicin was applied similarly to the TQ film. Wounds were covered using Tegaderm film (3M, Saint Paul, MN, USA). At each time point (Day 1, 2, 3, and 7) bacterial samples were collected by taking out the Tegaderm film from the wound site and kept in 2 mL microtube containing PBS. The tubes were then vortexed for 10 s to extract the bacteria. Different bacterial dilutions were made by adding 10 µL of the bacterial solution to the 990 µL of TSB solution and were plated on TSB agar plates. After overnight incubation at 37 °C bacteria were counted. The experiments were continued for 21 days. Wounds were visually monitored for local inflammatory reactions and photographed at Day 0, 3, 7, 10, 14, and 21 days. All the animals were euthanized at the end of day 21. The mice were observed at least once each day for signs of fatigue, stress, and aggressiveness. The mice were weighed at each time point.

### 2.18. Statistical Analysis

The cumulative amounts of TQ permeated per unit area were plotted against time. The flux was calculated by determination of the slope of the linear portion of the permeation profile. The % wound closure for each time interval was determined by the following formula and were calculated using NIH ImageJ software (NIH, Bethesda, MD, USA):

(1) $$\mathrm{wound}\;\mathrm{closure}\;\left(\%\right)\;=\frac{\mathrm{wound}\;\mathrm{diameter}\;\mathrm{on}\;\mathrm{day}\;0\;-\;\mathrm{wound}\;\mathrm{diameteron}\;\mathrm{on}\;\mathrm{day}\;n}{\mathrm{wound}\;\mathrm{diameter}\;\mathrm{on}\;\mathrm{day}\;0}\;\times 100\;$$

Results are reported as mean ± SD. The statistical analysis of the data was performed by using one-way analysis of variance (ANOVA) followed by post hoc Tukey HSD test and Student’s *t*-test. A *p*-value < 0.05 was considered statistically significant.

## 3. Results and Discussion

### 3.1. Characterization of the Compatibility of the Drug with Polymer

**Figure 2:**
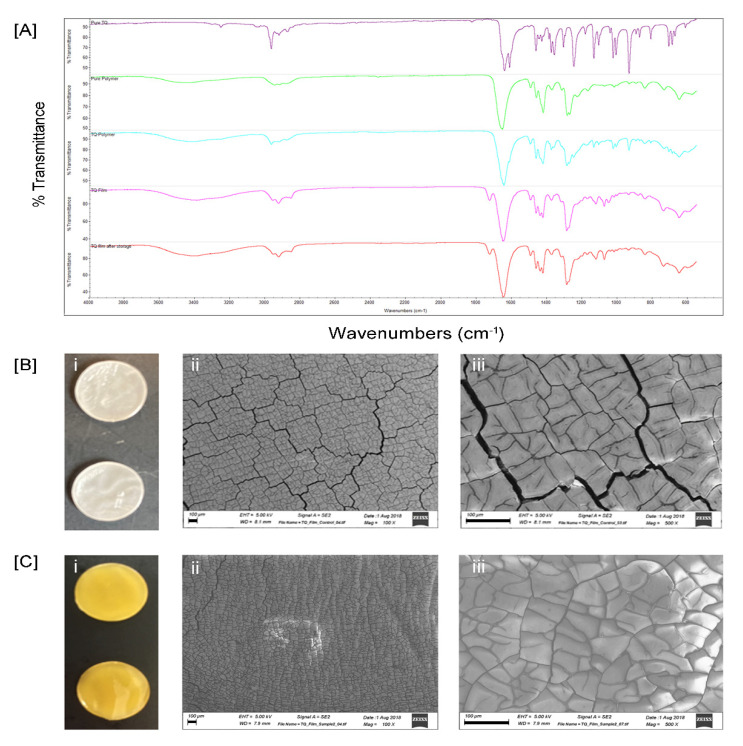
Physicochemical characterization of TQ films. (**A**) FTIR spectrum of TQ pure drug, PVP, physical mixture of drug and polymer, freshly prepared films containing drug and polymer, stored films containing drug and polymer; (**B**) Control films (i), Field emission scanning electron microscopic (FESEM) images showing the surface morphology of control film (ii–iii) at different magnifications (scale bar = 100 µm), and (**C**) TQ films (i), FESEM images showing the surface morphology of TQ films (ii–iii) at different magnifications (scale bar = 100 µm).

The FTIR analysis was employed to study the compatibility of the drug with the polymer used (Figure 2A). The samples were scanned in the region of 4000–400 cm^−1^. The IR spectral analysis of pure TQ showed that the major peaks were observed at wavenumbers 2967.30 (C-H stretching of aliphatic group), 1640.54 (C=C stretching), 1462.23, 1358.15 (C-H methyl rock), 1246.90, 1133.03, 1023.62 (C-H in-plane bend), 1006.33, and 933.08, confirming the purity of the drug (Figure 2A). A weaker band observed at a higher wavenumber (3253.95) corresponds to the stretching observed in the vinylic C-H in the C=C-H groups [30]. In the IR spectra of the physical mixture of TQ and PVP (Figure 2A) the major peaks of TQ were observed at wavenumbers 2966.73, 1644.49, 1461.38, 1374.84, 1248.05, 1133.32, 1023.30, 1006.10, and 933.43. Infrared spectra of the physical mixture of TQ and polymer showed all the characteristic peaks indicating the absence of any possible interaction between the drug and polymer. Therefore, it can be stated that the drug and polymer are compatible and can be formulated into films. The characteristic peaks of the drug can also be seen in TQ films of both freshly prepared and stored films (Figure 2A).

### 3.2. Physicochemical Characterization of Films

The results of the physicochemical studies are summarized in Table 1. The drug content in the prepared films was found to be 100% with a low standard deviation, indicating good uniformity in drug content. The thickness and weight variation of films are associated with the uniformity and accuracy of dosing [31]. Uniformity of thickness of each film and minimal weight variation was ensured as low standard deviation values were observed in the thickness of films and weight variation studies. The flatness study showed that the films had the same strip length before and after cutting, indicating 100% flatness. These data also indicate 0% constriction in the films meaning they could maintain a smooth surface when applied onto the skin. In other words, the films provided intimate contact with skin and hence better drug permeation. Folding endurance test results indicated that the films would not break and would maintain their integrity when folded. The low moisture uptake at laboratory ambient conditions protects the material from microbial contamination and avoids extra bulkiness of the films. The moderate moisture content of the prepared films could assist the formulation stability by preventing drying and brittleness. These results indicated that the polymeric combinations showed good film-forming properties and the process employed to prepare films in this study could produce films with uniform drug content and minimal film variability.

The surface morphology of the drug-loaded film was assessed using field emission scanning electron microscopy (FESEM) (Figure 2B,C). FESEM images were taken at different magnifications 100×, 500×, and 1000× to investigate the surface of films. At all magnifications, the film surface appeared smooth and compact. FESEM photograph of TQ film shows polymer networks inside the film and homogeneous dispersion of drug inside the polymer networks (Figure 2C).

### 3.3. Characterization of the TQ Hydrogels

The physicochemical properties of prepared TQ hydrogel formulations (F1–F10) are shown in Table 2. All the prepared hydrogels were light yellow and either opaque or clear. The homogeneity was determined by visual inspection and all the formulations were categorized into three groups (+++: very good—no lumps and smooth homogeneous texture; ++: good—no lumps and slightly uneven texture; +: not good—no lumps and inconsistent texture). Most of the formulations showed good homogeneity with no lumps and smooth homogeneous texture. pH values of the formulations were found in the range of 3.91–4.86. The relative standard deviation (% RSD) of prepared hydrogel formulations ranged from 0.12% to 0.34%. Good spreadability is one of the criteria for the gel as it shows the behavior of the gel when it comes out from the tube. It is the term that is used to indicate the extent of the area to which gel readily spreads on. It was observed that the spreadability of TQ hydrogels decreased by increasing the polymer concentration and the values were in the range of 12–21 mm.

Rheological properties of gel formulation provide important information regarding physical form, appearance, texture, and flow behavior [31]. The determination of the linear viscoelastic region (LVR) is used to understand the physical form or microstructure of the gel formulation as it presents critical stress, beyond which the sample may show significant structural breakdown. In this study, the oscillation stress sweep was used to obtain the LVR (the range of stress over which the elastic modulus G′ is independent from the applied stress amplitude). The mean stress value of 5 Pa was obtained from LVR and was used for other oscillation tests, such as frequency sweep.

The viscoelastic behavior of the prepared samples was obtained from the oscillation frequency sweep test. Elastic modulus (G′) and viscous modulus (G″) were determined as a function of frequency to measure the response of a system at constant stress amplitude (within LVR). The *G*′ of a sample will be large if the material is predominantly elastic or highly structured, as it reflects the energy stored per cycle and the solid-like component of the viscoelastic material, whereas G″ is a measure of the energy lost per cycle and reflects the fluid-like component.

**Figure 3:**
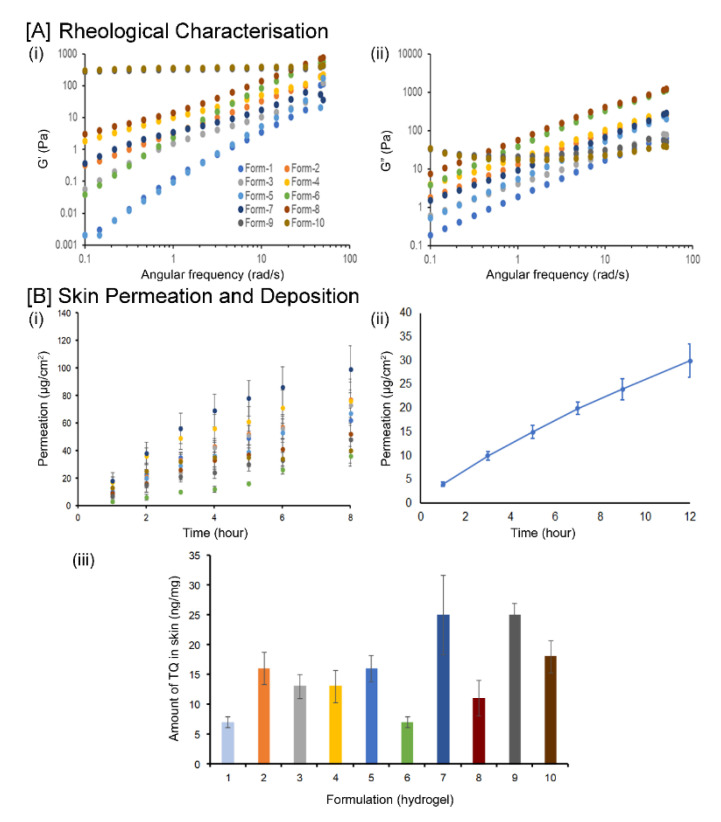
Rheological characterization of TQ hydrogel formulations (F1–F10). (**A**) Oscillation frequency sweep data. The elastic modulus (i); The viscous modulus (ii) were plotted against angular frequency. TQ permeation and skin deposition from film and gel formulations (**B**). TQ permeation profile for different hydrogel formulations (i). Time points were measured at 1, 2, 3, 4, 5, 6, and 8 h. Each point represents the five experiments; TQ permeation from film formulation across human cadaver skin (*n* = 5) (ii); Amount of TQ detected after 8 h in human cadaver skin (*n* = 5) using different TQ hydrogel formulations (iii).

In this experiment, the addition of Carbopol and Ultrez 10 showed a drastic increase in *G*′, whereas the addition of HPMC and HPC in formulation reduced *G*′. The order of increment in *G*′ was F10 > F9 > F4 > F3 > F1 (Figure 3A).

### 3.4. In Vitro Skin Permeation and Deposition

Penetration parameters of thymoquinone are summarized in Table 3. The results showed that the rank order for thymoquinone flux from each formulation are: F2 > F5 > F3 > F7 > F10 > F9 > F6 > F8 > F4 > F1. Figure 3B shows the thymoquinone permeation profile and amount of TQ detected after 8 h in human cadaver skin. It was observed that formulations 7 and 9 were able to retain more drugs in human cadaver skin compared to other formulations that might be useful in the treatment and management of wound infections.

### 3.5. Stability of TQ Films and Hydrogels

After storage, no significant changes in the color and texture of the film were observed. The drug content of the stored films was comparable and was within limits. Additionally, the FTIR spectra of stored film and freshly prepared films can be superimposed, indicating the stability of the films (Figure 2A). Hence, the film can be used after storage of two months without any loss of physical and chemical attributes. After storage for 8 months, hydrogels did not show any change in color and odor. Additionally, no phase separation occurred. The antibacterial study with the stored hydrogel showed similar efficacy like the marketed gentamicin cream against *S. aureus.* This indicated that the drug was stable in gels even after 8 months of storage and the gel formulations were physically and chemically stable.

### 3.6. Cytocompatibility

**Figure 4:**
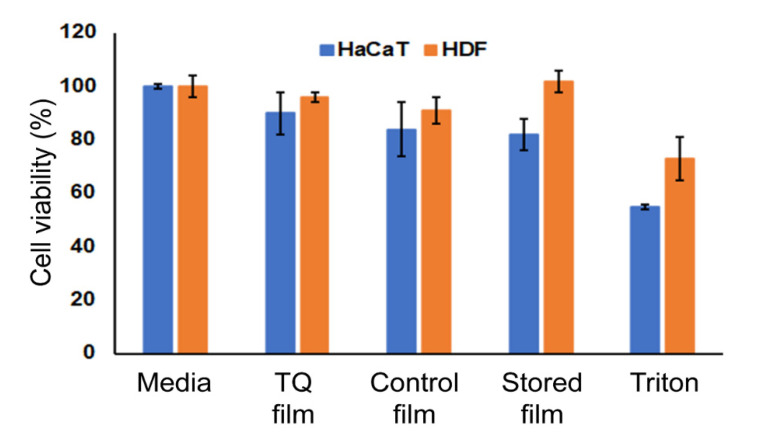
The cytocompatibility study of TQ film. Cell viability of TQ film with HDF and HaCaT cells using alamarBlue^®^ assay.

The viability of HDF and HaCaT cells at the presence of TQ film was analyzed using alamarBlue^®^ assay. The results of the test are shown in Figure 4. As can be seen from the graph, cell viability is not affected by the addition of TQ film in the media. TQ film showed 90% cell viability on HaCaT and 96% cell viability on HDF cell lines after 24 h of incubation. According to ISO 10993–5, 2009 [32] standards, it can be confirmed that the prepared samples were nontoxic because the cell viability is >80%.

### 3.7. TQ Films and Hydrogels Inhibit the Growth of S. aureus In Vitro and Ex Vivo

**Figure 5:**
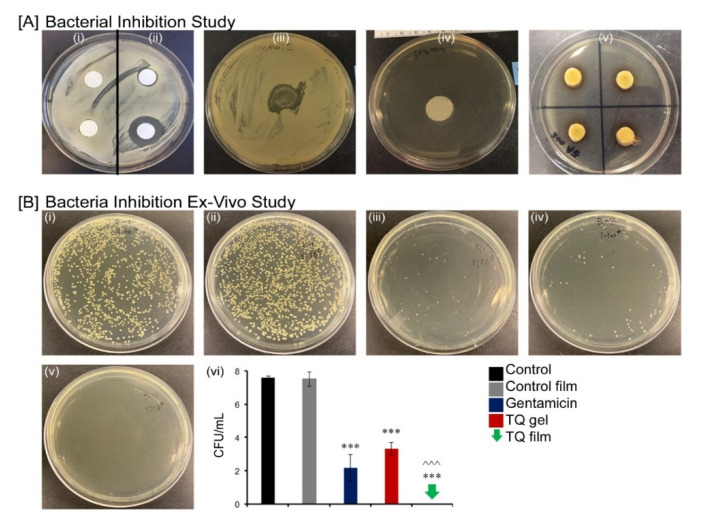
Bacterial inhibition study. (**A**) Inhibition of bacterial growth on agar plate by Control negative (i); Gentamicin positive control 50 µg/mL (ii) right upper and 500 µg/mL (ii) right lower; Control film (iii); TQ hydrogel (iv) and TQ film (v) against *S. aureus*; (**B**) Ex vivo antibacterial activity by Control (i); Control film (ii); Gentamicin sulfate USP, 0.1% marketed cream (iii); TQ hydrogel (iv); TQ film (v) and Log of bacterial reduction with different treatment groups (vi). Data represent of four replicates. *** *p* ≤ 0.001 and ^^^ *p* ≤ 0.05.

Before in vivo experiments, the in vitro antibacterial efficacy of TQ film was first validated along with ex vivo antibacterial activity. For this, the materials were inoculated with *S aureus* and their antibacterial efficacies against bacterial growth were assessed after 24-h incubation. Results from the in vitro study showed that the presence of gentamicin generated a zone of growth inhibition of *S. aureus* on the plate in a dosage-dependent manner (Figure 5A). However, there was no growth of bacteria in the presence of TQ films and hydrogels and suggested that complete inhibition of *S. aureus.* The complete absence of bacteria in the presence of TQ films was verified by three independent experiments. Furthermore, the results from the ex vivo antibacterial study showed complete eradication of bacteria using TQ film from human cadaver skin. Whereas, gentamicin cream and TQ hydrogel showed 5 and 4 log reduction of bacteria respectively (Figure 5B).

### 3.8. TQ Promotes the Wound Closure In Vitro

**Figure 6:**
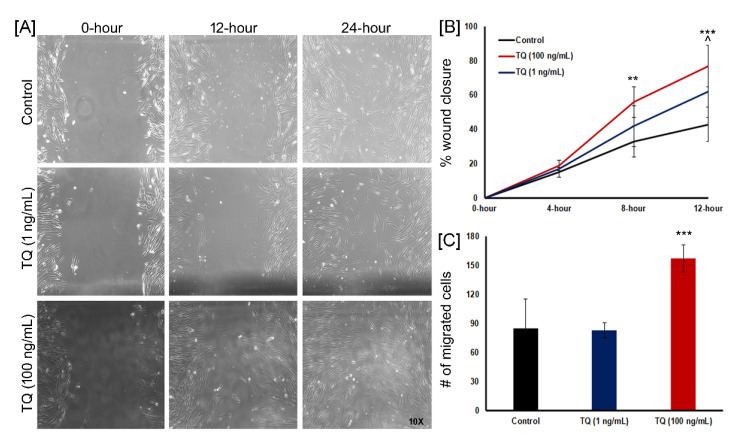
Effect of TQ treatment on the wound healing of human fibroblasts. (**A**) Representative micrographs (10×) from fibroblast cell migration including different treatment groups (Control, 1 ng/mL, and 100 ng/mL of TQ) showing the original wound and the wound after 12 h and 24 h; (**B**) Quantitative analysis of wound closure as a function of time. The wound area was determined as the wound area at a given time relative to the original wound area (*n* = 6). ** *p* < 0.01 and *** *p* < 0.001 (Control vs. 100 ng/mL) and ^ *p* < 0.05 (Control vs. 1 ng/mL); (**C**) Quantitative measurement of cell number migrating in the corresponding scratched wound areas at 24 h in the different treatment groups (*n* = 6). *** *p* < 0.001 (100 ng/mL vs. control and 1 ng/mL).

**Figure 7:**
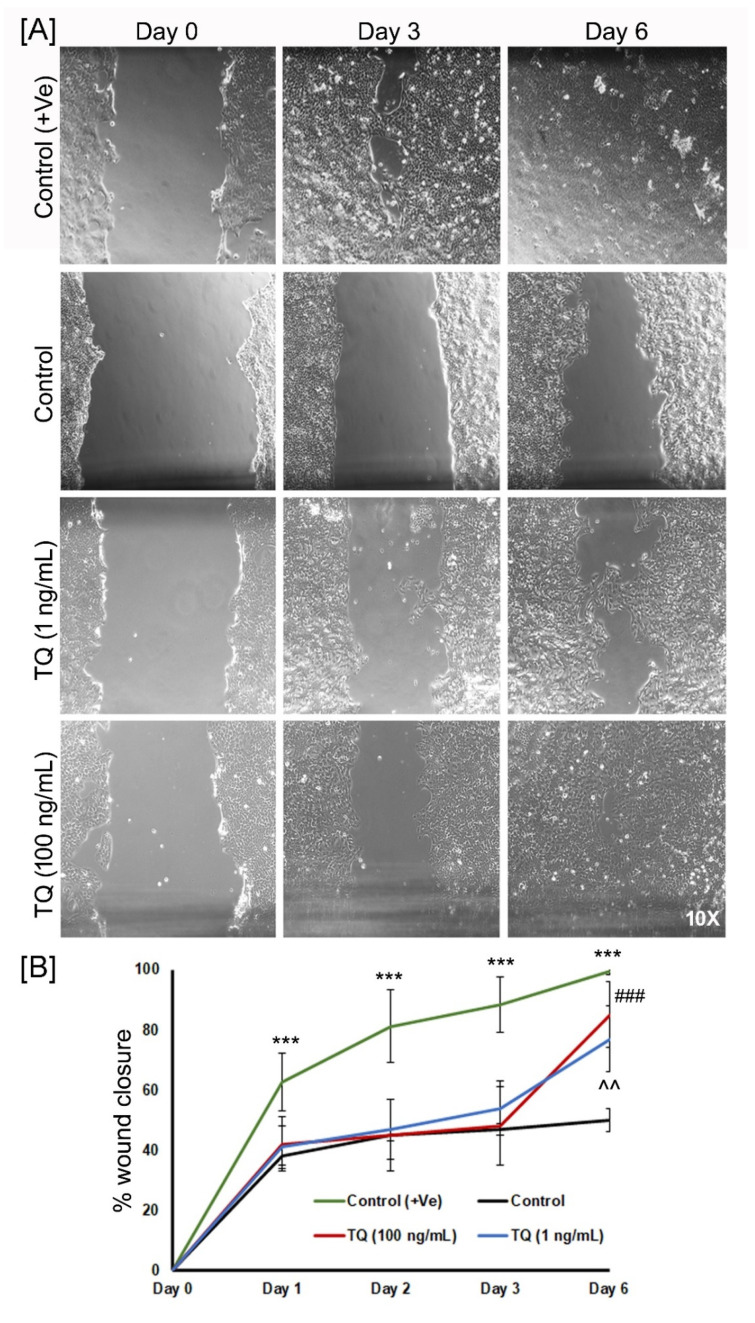
Effect of TQ treatment on the wound healing of keratinocytes using scratch assay. (**A**) Representative micrographs (10×) from Control (+Ve), Control, 1 ng/mL, and 100 ng/mL of TQ showing the original wound at day 0 and the wounds after 3 and 6 days of keratinocyte cell migration; (**B**) Quantitative analysis of wound closure as a function of time. The wound area was determined as the wound area at a given time relative to the original wound area at day 0 (*n* = 5–6). *** *p* < 0.001 (Control vs. Control + Ve), ^^ *p* < 0.01 (Control vs. 1 ng/mL) and ### *p* < 0.001 (Control vs. 100 ng/mL).

The in vitro scratch wound healing assay has been proven as a simple, valuable, and inexpensive technique to obtain first insights into how to plant-derived extracts or their isolated main active compounds can positively influence the formation of new tissue [33]. This assay has commonly been applied to measure cell migration, cell proliferation, and wound closure in response to test components. In this experiment, a “wound gap” in a cell monolayer of either HDF (Figure 6) or HaCaT (Figure 7) is created by scratching, and the ability of cells to migrate and repopulate the scratch area over time is monitored. The results of these experiments are shown in Figure 6 and Figure 7. It was found that TQ showed significant positive effects on wound healing activities of HDF and HaCaT cell lines. TQ (100 ng) showed significant wound closure activity with HDF compared to control (*p* < 0.05) (Figure 6A). 77% of wounds were closed with 100 ng TQ at 12 h followed by 100% wound closure at 24 h. On the other hand, only 43% of control wounds were closed at 12 h (Figure 6B). Additionally, at 4 h, 8 h, and 12 h, all the different concentrations of TQ (1 ng/mL and 100 ng/mL) showed increased wound closure activity compared to control, which suggests that cells migrated faster in the presence of TQ (Figure 6A,B). The number of fibroblasts migrated and counted at 24 h in the scratched area and migrated cells were higher in the 100 ng/mL TQ treated group (*p* = 0.0001) as compared to control and 1 ng/mL TQ groups (Figure 6C).

In the additional experiments using HaCaT cell line scratch assay, serum starvation (Control) slow down the cell migration as compared to positive Control without serum starvation (*p* = 0.0001) at all time points (Figure 7A,B). When these serum-starved groups treated with 1 ng/mL and 100 ng/mL of TQ led to increased wound closure activity compared to the control at day 6 (*p* < 0.01 and *p* < 0.001, respectively). Rapid wound closure activity was observed between day 3 and day 6 of TQ treated groups.

### 3.9. Anti-Bacterial and Wound Healing Activity of TQ Film In Vivo

**Figure 8:**
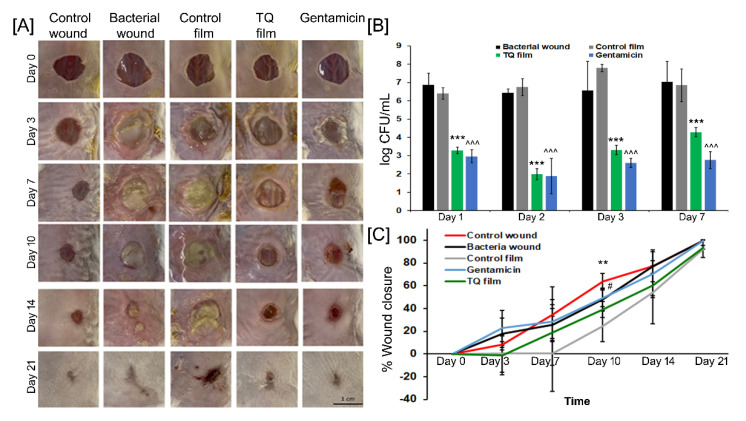
Antibacterial effects of TQ films on full-thickness skin wounds infected with *S. aureus*. (**A**) Photographs of skin wounds with different treatments over 21 days. The wounds were infected with bacteria as shown above as a pale biofilm in the bacteria wound group and other related groups. Scale bar = 1 cm. (**B**) Log of bacterial reduction at each timepoint up to 7 days assessed in different experimental groups. *** *p* < 0.001 (Bacterial wound vs. TQ Film) and ^^^ *p* < 0.001 (Bacterial wound vs. Gentamicin). (**C**) Percentage of wound closure as a function of time in all experimental groups at different time points post-wounding and treatments. ** *p* < 0.01 (Control wound vs. Control Film) and # *p* < 0.05 (Control Film vs. Gentamicin).

The photographs of the wounds at different time points post-wounding are shown in Figure 8A which suggests the wound infection and antibacterial activity of TQ film. For this assay, bacterial samples were collected at day 1, 2, 3, 7, and sample analysis of the wound site showed significant (*p* < 0.001 all-time points versus bacterial control and control film) bacterial reduction using both TQ film and gentamicin groups compared to the bacterial wound and control film groups (Figure 8B). After the bacterial reduction seen in the TQ film and gentamicin groups, we carry forward these animals for wound closing timepoint up to day 21 (Figure 8A,C) to study the wound healing effects of TQ films. Although all the wounds closed by day 21 except the bacterial infection group that shows the effect of bacterial infection and inhibition of wound closure. There is no significant effect of TQ on wound healing but the gentamicin and control group (without bacteria) attain significance at day 10 compared to the control film (Figure 8C). Therefore, future research should be conducted to find out the appropriate dosage regimen (formulation type, TQ concentration in the formulation, dosing interval, length of treatment) that would be needed to bring in the beneficial effect of TQ in the faster wound closing. Based on the experimental results, we know that TQ is a strong strategy to disinfect skin wounds. Also, manipulation in the TQ formulation and structure needed to design a faster wound closing TQ formulation. Future research should also examine the usefulness of other dosage forms of TQ such as creams, gels, and topical spray formulations in wound closure activities.

Previous studies have shown that TQ exhibits a selective antibacterial effect against multiple bacteria, particularly Gram-positive strains with low MICs (minimum inhibitory concentration) values [34,35]. Mohammed et al. demonstrated that free rotation bonds present in TQ might render this compound very flexible for forming several conformations [36]. This behavior of the bond in the CH3 group might further enable TQ to alter their shapes to easily correspond to the bacteria and enter or cross their boundary and eventually kill them [36]. More study needs to be done to precisely determine the comprehensive mechanism by which TQ exerts its antimicrobial properties.

## 4. Conclusions

Open wounds are prone to bacterial infection and if not treated early enough may also provide an entry point for microbes that cause systemic infections. Additionally, infected wounds heal less rapidly since infection at the wound site can produce toxins that can further kill the regenerating cells and can delay the progression of wound healing. Several topical and oral antibiotics are presently being used to treat wound infections in humans. However, due to their adverse side effects and the presence of antibiotic-resistant organisms, there is a need to investigate alternative sources such as bioactive compounds of plant origin for their antibacterial activity to offer an innovative treatment strategy. The use of TQ films to treat infected wounds in this study have shown a potential disinfectant strategy for chronically infected wounds such as diabetic foot ulcer and ulcers after spinal cord injury, as TQ films have been shown to exhibit significant activity against *S. aureus*.

In this study, novel TQ loaded polymeric films and hydrogels were developed using different polymers. The application of this TQ containing films and hydrogels showed in vitro TQ skin permeation and strong antibacterial activity against *S. aureus* since no bacterial growth was observed with TQ loaded film and hydrogel formulations. In vitro scratch wound healing assay revealed wound closure activity of TQ. Moreover, the preclinical mouse model of *S. aureus* wound infection demonstrated a significant reduction in the bacterial population using TQ films. Based on these data we can conclude that significant bacterial reduction by TQ film has the potential to provide a new treatment strategy especially for those patients who have developed resistance towards the commonly used antibacterial agents. TQ/PVP films and hydrogels developed in this study have the potential for the treatment and management of wound and *S. aureus* related bacterial skin and wound infections. However, further research (in vitro and in vivo) is needed to confirm these novel findings for enhancing skin wound healing. Some potential areas would include dosing studies as well as alternative user-friendly dosage forms such as sprays and foams.

## Figures and Tables

**Table 1:** Physicochemical properties of TQ films.

Physicochemical Parameters	TQ Polymeric Films *
Drug content (%)	100 ± 6.4
Thickness (mm)	1.17 ± 0.04
Weight variation (mg)	82.04 ± 1.9
Flatness (%)	100 ± 0.0
Folding endurance	68 ± 2.38
Moisture content (%)	14.12 ± 0.42
Moisture uptake (%)	2.26 ± 0.47

* Data show mean of five determinations with ± standard deviation.

**Table 2:** Physicochemical properties of TQ topical hydrogel formulations (F1–F10).

Formulations	Color	Homogeneity	pH	Spreadability (mm)	Content Uniformity (% RSD)
1	Light yellow, Opaque	+	3.91	21	0.24
2	Light yellow, Opaque	++	3.99	15	0.15
3	Light yellow, Opaque	+	4.18	17	0.04
4	Light yellow	+	4.26	15	0.21
5	Light yellow, Clear	+++	4.06	17	0.22
6	Light yellow, Clear	+++	4.12	12	0.25
7	Light yellow, Opaque	+++	4.21	16.5	0.12
8	Light yellow, Opaque	+++	4.34	12	0.25
9	Light yellow, Clear	+++	4.86	13	0.34
10	Light yellow, Clear	+++	4.71	12	0.26

+: not good; ++: good; +++: very good.

**Table 3:** Penetration parameters of thymoquinone through human cadaver skin (*n* = 5) after 8 h.

Formulation	Q at 8 h (µg/cm^2^)	TQ Flux (µg/cm²/h)
1	62 ± 9	5.38 ± 0.3
2	77 ± 13	9.56 ± 1.0
3	73 ± 11	7.87 ± 0.9
4	76 ± 16	5.59 ± 0.7
5	67 ± 15	9.19 ± 2.0
6	36 ± 7	6.70 ± 1.8
7	99 ± 17	7.11 ± 1.6
8	52 ± 9	6.24 ± 1.1
9	48 ± 10	6.89 ± 1.4
10	40 ± 9	7.1 ± 2.3

Q—cumulative amount of thymoquinone penetrated per cm^2^ of skin at 8 h (*n* = 5).

## Supplementary Materials

The following are available online at https://www.mdpi.com/2227-9059/8/10/386/s1, Table S1. Composition of TQ topical hydrogels F1–F10 (% *w*/*w*).
