# Supplementary material for: Thymoquinone-Loaded Polymeric Films and Hydrogels for Bacterial Disinfection and Wound Healing
Source: Biomedicines. 2020 Sep 28;8(10):386. doi: 10.3390/biomedicines8100386 (PMC7600314; doi:10.3390/biomedicines8100386)
Supplement: Supplementary file 1 [file biomedicines-08-00386-s001.pdf]

Table S1. Composition of TQ topical hydrogels F1–F10 (% *w*/*w*).

Ingredients	F1	F2	F3	F4	F5	F6	F7	F8	F9	F10
TQ	0.2	0.2	0.2	0.2	0.2	0.2	0.2	0.2	0.2	0.2
XG	-	-	0.2	0.2	-	-	0.2	0.2	-	-
HPMC	4	6	4	6	-	-	-	-	-	-
HPC	-	-	-	-	12	18	12	18	-	-
Carbopol	-	-	-	-	-	-	-	-	1	-
Ultrez-10	-	-	-	-	-	-	-	-	-	1
PG	10	10	10	10	10	10	10	10	10	10
DiPG	10	10	10	10	10	10	10	10	10	10
EtOH	5	5	5	5	5	5	5	5	5	5
Benzoic Acid	0.1	0.1	0.1	0.1	0.1	0.1	0.1	0.1	0.1	0.1
DI water to	100	100	100	100	100	100	100	100	100	100
